# Weight loss-induced changes in adipose tissue proteins associated with fatty acid and glucose metabolism correlate with adaptations in energy expenditure

**DOI:** 10.1186/s12986-015-0034-1

**Published:** 2015-10-24

**Authors:** Stefan G. J. A. Camps, Sanne P. M. Verhoef, Nadia Roumans, Freek G. Bouwman, Edwin C. M. Mariman, Klaas R. Westerterp

**Affiliations:** Department of Human Biology, Nutrition and Toxicology Research Institute Maastricht, Maastricht University, PO Box 616, 6200 MD Maastricht, The Netherlands

**Keywords:** Energy expenditure, Physical activity, Body composition, Adipose tissue, Glucose metabolism, Fatty acid metabolism

## Abstract

**Background:**

Energy restriction causes adaptations in energy expenditure (total-,TEE; resting-,REE; activity induced-,AEE).

**Objective:**

To determine if changes in the levels of proteins involved in adipocyte glucose and fatty acid metabolism as indicators for energy deficiency are related to adaptations in energy expenditure during weight loss.

**Methods:**

Forty-eight healthy subjects (18 men, 30 women), mean ± SD age 42 ± 8 y and BMI 31.4 ± 2.8 kg/m^2^, followed a very low energy diet for 8 wk. Protein levels of fatty acid binding protein 4 (FABP4), fructose-bisphosphate aldolase C (AldoC) and short chain 3-hydroxyacyl-CoA dehydrogenase (HADHsc) (adipose tissue biopsy, western blot), TEE (doubly labeled water), REE (ventilated hood), and AEE were assessed before and after the 8-wk diet.

**Results:**

There was a positive correlation between the decrease in AldoC and the decrease in TEE (*R* = 0.438, *P* < 0.01) and the decrease change in AEE (*R* = 0.439, *P* < 0.01). Furthermore, there was a negative correlation between the increases in HADHsc and the decrease in REE (*R* = 0.343, *P* < 0.05).

**Conclusion:**

The decrease in AldoC correlated with the decrease in AEE, which may be explained by a decreased glycolytic flux. Additionally, the change in HADHsc, a crucial enzyme for a step in beta-oxidation, correlated with the adaptation in REE.

**Trial registration:**

Clinical Trial Registration Number: NCT01015508 at clinicaltrials.gov

## Introduction

The increasing prevalence of obesity and its comorbidities is one of the major health problems in our modern world [1]. Although weight loss strategies target both sides of the energy balance, intake and expenditure, the success of long-term weight loss maintenance is low [2, 3]. Adipose tissue is an important energy storing and releasing tissue and to fulfill this role adipocytes need to respond quickly to variations in the demand. Many studies have linked obesity to metabolic processes on a whole-body level, like reduced fat oxidation, as well as inside the adipocyte like a reduced metabolism of long chain fatty acids [4–7]. Studies on weight loss indicate that energy restriction results in changes in the expression of genes involved in lipid, carbohydrate and energy metabolism in adipose tissue [8–13]. During energy restriction, the limiting glucose availability must be compensated by an increased mitochondrial fatty acid oxidation to preserve blood glucose levels and supply glucose-dependent tissues with sufficient energy, such as the brain or red blood cells [14].

The biological response to weight loss is causing the susceptibility to weight regain as reviewed by MacLean et al. [15]. Mariman summarized this response as a network of adaptations with an energy gap promoting weight regain and physiological changes resulting in resistance for further weight loss [16]. Two of these adaptations are decreases in energy expenditure in response to energy restriction, which can limit weight loss and could be important factors that compromise the maintenance of a reduced body weight. Firstly, studies performed in lean and obese subjects have shown significant reductions in resting energy expenditure (REE) during and shortly after weight loss, to values below predictions based on weight loss and body composition changes [17–22]. The decrease in REE beyond what can be predicted by the loss of fat-free mass (FFM) and fat mass (FM) is defined as adaptive thermogenesis. Secondly, several studies demonstrated a decrease in physical activity and activity induced energy expenditure (AEE) as a result of weight loss [23–28].

Linking the physiological adaptations to the glucose and fatty acid metabolism will give further insight in the underlying metabolic responses. For this, changes in the levels of proteins involved in glucose and fatty acid metabolism were measured in adipose tissue before and after an 8-wk very low energy diet. Previously, Verhoef et al. showed results of such changes with parameters of adiposity during weight loss and maintenance [11]. Here, in the same subject population, the aim of the study was to determine whether such protein changes are associated with adaptations in energy expenditure. As target proteins, fructose-bisphosphate aldolase C (AldoC), short chain 3-hydroxyacyl-CoA dehydrogenase (HADHsc) and fatty acid binding protein 4 (FABP4) were chosen. In all participants of a weight loss-maintenance study, two isoforms of AldoC consistently decreased in abundance in the adipose tissue during energy restriction [11, 13]. Although CPT1a is regarded to be the overall rate-limiting factor for mitochondrial uptake and oxidation of fatty acids, HADHsc has been shown to be involved in the rate-limiting acyl-CoA dehydrogenase step of beta-oxidation [29]. This was later confirmed for oxidation of unsaturated fatty acids with odd-numbered double bonds [30]. In a weight loss-maintenance intervention, HADHsc was up-regulated in the adipose tissue of every participant due to energy restriction [12]. FABP4 is a general transporter of fatty acids in the adipocyte.

### Subjects and methods

#### Subjects

Forty-eight healthy subjects (30 women and 18 men), mean + SD age of 42 ± 8 y and mean ± SD body mass index (BMI) of 31.4 ± 2.8 kg/m^2^, were recruited by advertisements in local newspapers and on notice boards at the university. They underwent an initial screening that included measurement of body weight and height and the completion of a questionnaire on general health. All were in good health, not using medication (except for contraception), nonsmokers and at most moderate alcohol consumers. They were weight stable as defined by a weight change <5 kg for at least 3 months prior to the study. The study was conducted according to the guidelines laid down in the Declaration of Helsinki and procedures were approved by the Ethics Committee of the Maastricht University Medical Centre. Written informed consent was obtained from all participants. This trial was registered at clinicaltrials.gov as NCT01015508.

### Study design

The study covered an 8-wk period of a very low energy diet (VLED). Subjects came to the university for measurements on two occasions: the day before the start of the diet (baseline) and 8 wk after the start of the diet (end of the diet). The protocol included an overnight stay at the university followed by the collection of blood, measurement of REE and body composition from 08.00 in the morning onwards in the fasting state. Two wk prior to each measurement day, subjects received a subject-specific dose of doubly labeled water to measure total energy expenditure (TEE) over the following 14 days.

### Diet

The weight loss diet (Modifast; Nutrition et Santé Benelux, Breda, The Netherlands) was followed for a period of 8 wk. The diet was a protein-enriched formula that provided 2.1 MJ/day (51.9 grams of protein, 50.2 grams of Carbohydrates and 6.9 grams of Lipids) and a micronutrient content, which meets the Dutch recommended daily allowance. The VLED was provided to the subjects as sachets with powder. Each sachet represented one meal and 3 sachets were consumed every day. In addition to the provided meal-replacements, subjects were allowed to eat vegetables when feeling hungry. Subjects were instructed to mix the powder with the amount of water indicated on the packages and were advised to drink water sufficiently throughout the diet period.

### Body composition

Height was measured at screening to the nearest 0.1 cm with the use of a wall-mounted stadiometer (model 220; Seca, Hamburg, Germany). Body composition was determined according to Siri’s three-compartment model based on body weight, body volume and total body water. Body weight was measured using a calibrated scale (Life Measurement Corporation, Inc, Concord, CA, USA). Body volume was measured via air-displacement plethysmography with the BodPod System (Life Measurement Corporation, Inc, Concord, CA, USA) [31, 32]. Total body water was determined using deuterium dilution during the preceding night, according to the Maastricht protocol [33]. BMI was calculated by dividing body weight by height squared (kg/m^2^).

### Resting energy expenditure

At 0800 h in the morning, after the overnight stay at the university, subjects slowly walked to a separate room where they rested on a bed for 30 min, followed by 30 min of measuring their REE in the supine position using an open-circuit ventilated hood-system [34]. Gas analyses were performed with a paramagnetic oxygen analyzer (Servomex, type 1158, Crowborough, East Sussex, UK) and an infrared carbon dioxide analyzer (Servomex, type 1520, Crowborough, East Sussex, UK) while flow was kept at a constant rate of 80 l/min and additionally measured as described by Schoffelen et al. [35]. The within individual coefficient of variation for this system is 3.3 % ± 2.1 [34]. Calculation of REE from measured oxygen consumption and carbon dioxide production was based on Brouwer’s formula [36].

In addition to measuring REE with the ventilated hood system (REEm), REE was predicted (REEp) with the equation: REEp (MJ/d) = 0.024 x fat mass (kg) + 0.102 x fat free mass x (kg) + 0.85 [37]. Since fat mass (FM) and fat free mass (FFM) are used to calculate REEp, the equation can be used independently for gender. Adaptive thermogenesis was calculated as REEm divided by REEp [22].

### Total en activity induced energy expenditure

Estimates of TEE were measured over two wk intervals with the doubly labeled water method according to the Maastricht protocol [33]. The doubly labeled water method is considered the gold standard for measuring TEE under field conditions [38]. On the evening of day 1, shortly after the collection of a background urine sample, subjects drank a weighed amount of ^2^H_2_^18^O such that baseline levels were increased by 100–150 ppm for ^2^H and 200–250 ppm for ^18^O. Subsequently, urine samples were collected in the morning of day 2 (second voiding), day 8 and day 14 and in the evening of day 1, 8 and 13. The doubly labeled water method gives precise and accurate information on carbon dioxide (CO_2_) production. CO_2_ production was subsequently converted to TEE with the use of the energy equivalent of CO_2_, which can be calculated with additional information on the substrate mixture being oxidized [39]. The energy equivalent at baseline was calculated based on a normal Western diet with a mixed macronutrient composition and energy respectively for 55 % from carbohydrate, 30 % from fat and 15 % from protein. At the end of the diet, the energy equivalent of CO_2_ was based on the consumption of the Modifast diet, the actual loss of fat mass and fat free mass and additional energy intake. The additional food intake was the calculated compensation for the difference between weight loss and the expected weight loss based on the consumption of the Modifast diet alone, with the assumption of 1 kg weight change to be equivalent to 30 MJ [40]. The additional food intake was also assumed to be a normal mixed diet.

The indicated method to estimate AEE is the doubly labeled water method for the measurement of TEE in combination with a measurement of REE [41]. At baseline, AEE was calculated as (0.9 X TEE) – REE, assuming diet induced thermogenesis (DIT) to be 10 % of TEE, which is based on a normal mixed diet [42] and DIT values for the separate macronutrients to be 10 % for carbohydrate, 3 % for fat and 25 % for protein. At the end of the diet, the percentage DIT was calculated based on the intake of the Modifast diet and the additional food intake with a normal mixed composition, which accounted for the difference between the expected weight loss and the real weight loss. DIT was calculated to be 8 % of the TEE at the end of the diet; therefore AEE was calculated as (0.92 X TEE) – REE.

### Western blot analysis

Three proteins involved in glucose and fatty acid metabolisms were selected and measured in adipose tissue by Western blotting. Fructose-bisphosphate aldolase C (AldoC), fatty acid binding protein 4 (FABP4) and short chain 3-hydroxyacyl-CoA dehydrogenase (HADHsc).

Abdominal subcutaneous adipose tissue biopsies (approximately 1.5 g) were obtained by needle liposuction under local anaesthesia (2 % lidocaine, Fresenius Kabi BV, The Netherlands) after an overnight fast, before and after the diet. Samples were rinsed in sterile cold saline, frozen in liquid nitrogen and stored at −80 °C until protein isolation.

Frozen adipose tissue was grinded in a mortar and the powder was dissolved in 200 μl of 8 M urea, 2 % CHAPS, 65 mM DTT per 100 mg powder. The homogenate was vortexed for 5 min and centrifuged for 30 min at 14000 rpm and 10 °C. The supernatant containing the adipose tissue proteome was carefully collected and aliquots were stored at −80 °C. Protein concentrations were determined by a Biorad Bradfort-based protein assay [43].

Samples with equal amount of protein were run on a 12 % SDS polyacrylamide gel (180 V, Criterion Cell; Biorad, Hercules, CA) and then transferred (90 min, 100 V, Criterion blotter; Biorad) to 0.45-mm nitrocellulose membranes. After Ponceau S staining and destaining with demineralized water, membranes were blocked in 5 % bovine serum albumin (BSA) in Tris-buffered saline containing 0.1 % Tween 20 (TBST) for AldoC, and in 5 % nonfat dry milk powder in TBST for FABP4 and HADHsc for 1 h. Thereafter, the blots were incubated with the primary antibodies against AldoC (1:250 dilution, Santa Cruz sc-12066), FABP4 (1:1000 dilution, Cayman 10004944) and HADHsc (1:500 dilution, Santa Cruz sc-74650) in 5 % BSA-TBST (AldoC) or 5 % nonfat dry milk powder TBST (FABP4, HADHsc) overnight at 4 °C on a shaker. Thereafter, the blots were washed three times for 10 min in TBST, and then incubated with 1:10000 dilution of the horseradish peroxidase-conjugated secondary antibody (DAKO) in 5 % BSA-TBST, 0.5 % nonfat dry milk powder TBST or 5 % nonfat dry milk powder TBST for 1 h. The blots were washed three times for 10 min in TBST. A CCD camera (XRS-system, Biorad) was used to detect immunoreactive bands using chemiluminescent substrate (SuperSignal CL; Pierce). The quantification was performed with the program Quantity One version 4.6.5 (Biorad). Blots were normalized to beta-actin (1:1000 dilution, Santa Cruz sc-47778) to correct for differences in protein loading.

### Biochemical analysis

Blood was collected into EDTA-containing tubes and centrifuged (1000 × g, 10 min, 4 °C), and the plasma was immediately frozen in liquid nitrogen and stored at −80 °C until analysis. Plasma glucose concentration was analyzed enzymatically on a Cobas Mira automated spectrophotometer (Roche Diagnostica). Plasma insulin was measured with a double-antibody radioimmunoassay (Insulin RIA 100, Kabi-Pharmacia, Uppsala, Sweden).

### Calculations and statistical analysis

A paired t-test (two-tailed distribution) was carried out to determine possible differences between mean values. Regression analysis and Spearman Rho's correlation coefficients were calculated for associations between parameters. One-way repeated measures ANOVA were used to compare the results across 0 and 8 wk, with gender as covariate. Significance was defined as *P* < 0.05. The power calculation was based on a weight loss study, in which a significant 2-fold increase in HADHsc level was measured with Western blotting during weight loss in 8 obese subjects [12]. With an α of 0.05, beta of 0.10, mean change of 0.285, and standard deviation of 0.223 for HADHsc, and taking into account an expected success-rate of 20 % during weight maintenance and a dropout rate of 15 %, at least 29 subjects needed to be included at the start of the study. The data were analyzed using SPSS 20.0 (SPSS, Inc., Chicago, IL, USA). All data are presented as mean and standard deviation (SD).

## Results

### Body composition

After the 8 wk of VLED, weight loss was on average 9.9 ± 4.1 kg (*P* < 0.001) consisting of 7.9 ± 3.3 kg of fat mass (FM) and 2.0 ± 2.2 kg of fat free mass (FFM) (Table 1). Subjects lost on average 10.7 ± 4.1 % (*P* < 0.001) of the starting weight. FM decreased from 41.8 ± 6.3 % to 37.3 ± 7.5 % (*P* < 0.001). The data showed a large inter-individual variation in weight loss. The variation in weight loss was not explained by different levels of physical activity at baseline or 8 wk.

**Table 1:** Subject characteristics (mean ± SD) at baseline and after 8 wk on a very low energy diet

	Pre-WL (0 wk)	Post-WL (8 wk)	*P*-value
Weight (kg)	91.9 ± 13.1	82.0 ± 11.2	<0.001
BMI (kg/m^2^)	31.4 ± 2.8	28.3 ± 3.0	<0.001
FM (kg)	36.3 ± 7.3	28.4 ± 6.7	<0.001
FFM (kg)	56.1 ± 10.3	54.0 ± 9.5	<0.001
TEE (MJ/d)	12.65 ± 2.08	10.38 ± 1.95	<0.001
REE (MJ/d)	7.52 ± 0.98	6.74 ± 0.85	<0.001
AEE (MJ/d)	4.42 ± 1.47	3.27 ± 1.24	<0.001
Glucose (mmol/l)	4.9 ± 0.5	4.8 ± 0.6	0.322

*BMI* body mass index, *FM* fat mass, *FFM* fat free mass, *TEE* total energy expenditure, *REE* resting energy expenditure, *AEE* activity induced energy expenditure

### Energy expenditure

TEE decreased significantly from 12.65 ± 2.08 MJ/d at baseline to 10.38 ± 1.95 MJ/d (*P* < 0.001) after the VLED; a decrease of 17.5 ± 11.9 % (*P* < 0.001).

REE decreased significantly from 7.54 ± 1.05 MJ/d at baseline to 6.70 ± 0.87 MJ/d (*P* < 0.001) after 8 wk of VLED, which is a decrease of 10.8 ± 6.6 % (*P* < 0.001). The decrease was explained by the reduced body weight and adaptive thermogenesis in response to the diet.

AEE decreased from 4.42 ± 1.47 MJ/d at baseline to 3.27 ± 1.24 MJ/d after 8 wk of energy restriction (*P* < 0.001); a decrease of 22.8 ± 28.7 % (*P* < 0.001) (Table 1).

### Blood glucose

Fasting glucose concentration at baseline was on average 4.9 ± 0.5 mmol/l and was not different after weight loss (4.8 ± 0.6 mmol/l).

### Proteins

**Fig. 1:**
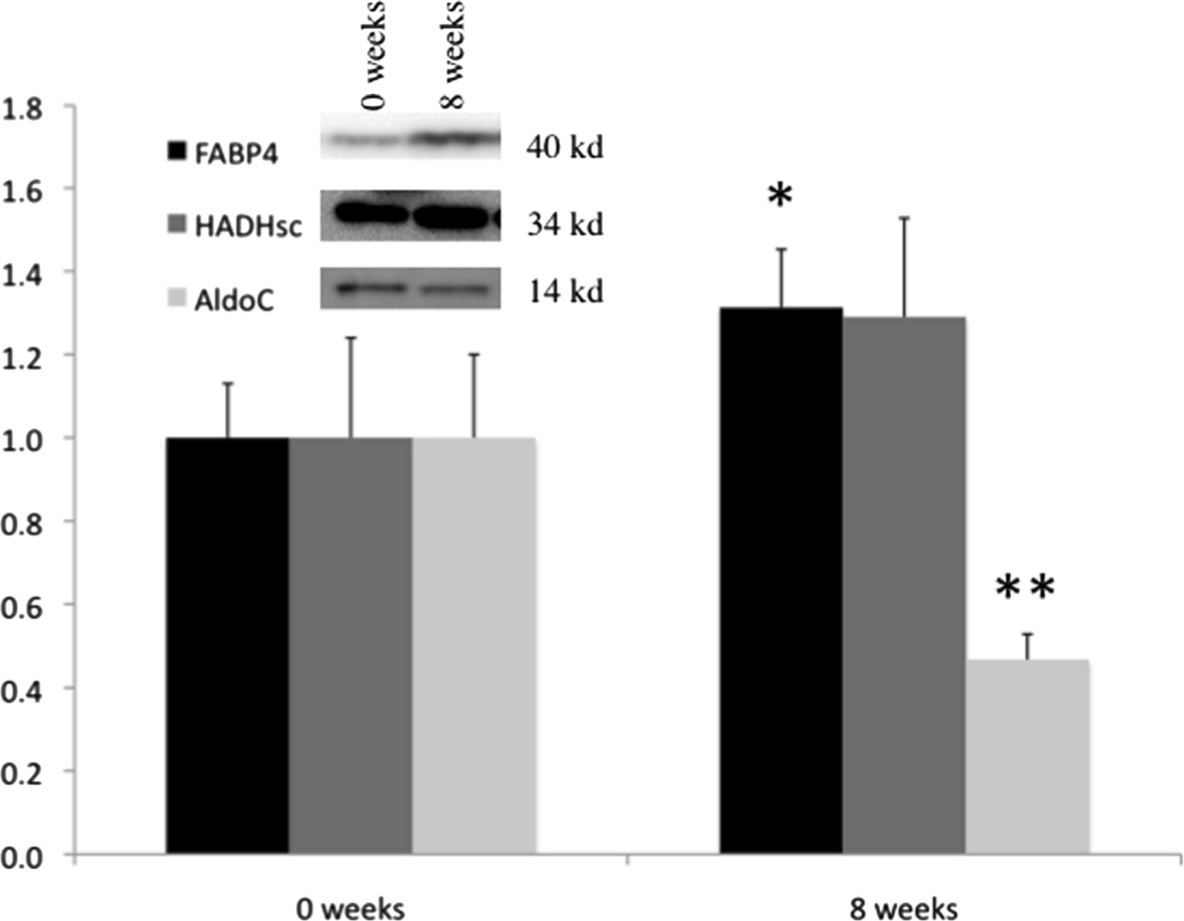
Fold changes of post-WL (8 wk) compared to pre-WL (0 wk). Fold changes are obtained by dividing the average spot intensity of the post-WL group by that of the pre-WL group. Protein levels of FABP4, HADHsc and AldoC were analyzed using Western blotting. Representative results of 3 independent experiments are shown; Beta-actin was used as an internal control to ensure equal loading in all lanes of the gel (results not shown). *P < 0.05, **P < 0.001, FABP4, Fatty acid binding protein 4, HADHsc, Short chain 3-hydroxyacyl-CoA dehydrogenase, AldoC, Fructose-bisphosphate aldolase C

Fatty acid binding protein 4 (FABP4) increased by 31.3 ± 87.0 % during VLED (*P* < 0.05). Fructose-bisphosphate aldolase C (AldoC) decreased significantly with 53.2 ± 37.0 % during the diet (*P* < 0.001). There was no significant change in short chain 3-hydroxyacyl-CoA dehydrogenase (HADHsc) during the VLED-period (Fig. 1).

After 8 wk, the change in FABP4 was significant negatively correlated with the change in FM (*R* = −0.35, *P* < 0.05), while the change in AldoC was significant positively correlated with the change in FFM (*R* = 0.52, *P* < 0.001) (Table 2).

**Table 2:** Spearman Rho’s correlation coefficients of changes in protein levels with changes in body composition and energy expenditure parameters. Increased (+) and decreased (−) parameters are indicated in the first column

	FABP4	HADHsc	AldoC
FABP4 (+)		0.38*	0.47**
HADHsc	0.38*		0.46**
AldoC (−)	0.47**	0.46**	
FM (−)	−0.35*		
FFM (−)			0.52***
REE (−)		0.34*	
AEE (−)			0.44*
TEE (−)			0.44*

*P < 0.05, **P < 0.01, ***P < 0.001, *FABP4* fatty acid binding protein 4, *HADHsc* short chain 3-hydroxyacyl-CoA dehydrogenase, *AldoC* fructose-bisphosphate aldolase C, *FM* fat mass, *FFM* fat free mass, *REE* resting energy expenditure, *AEE* activity induced energy expenditure, *TEE* total energy expenditure

After weight loss, there was a significant positive correlation between the change in AldoC and the percentage change in TEE (*R* = 0.44, *P* < 0.01); more specifically there was a significant positive correlation between the change in AldoC and the percentage change in AEE (*R* = 0.44, *P* < 0.01). Furthermore, there was a significant positive correlation between the change in HADHsc and adaptive thermogenesis in REE after the VLED (*R* = 0.34, *P* < 0.05).

After 8 wk, there were positive correlations between the changes in FABP4 and HADHsc (*R* = 0.38, *P* < 0.05), between FABP4 and AldoC (*R* = 0.47, *P* < 0.01) and HADHsc and AldoC (*R* = 0.46, *P* < 0.01) (Table 2).

## Discussion

Measuring proteins involved in glucose and fatty acid metabolism before and after an 8-wk VLED reflects the metabolic adaptations occurring in adipose tissue linked to energy expenditure. More specifically, the decrease of AldoC, an enzyme of glycolysis, is correlated with the decrease in AEE, and the non-significant change HADHsc, a crucial enzyme for mitochondrial beta-oxidation, is negatively correlated with the adaptation in REE. Furthermore, there is a correlation between the increase in FABP4, the intracellular fatty acid transporter, and the decrease in fat mass, and a correlation between the decrease in AldoC and the decrease in fat free mass. FABP4, AldoC and HADHsc are all positively correlated.

The increased FABP4 after the VLED weight loss is in accordance with previous results in obese subjects [11, 12, 44]. It is in line with an elevation in intracellular trafficking of fatty acids, which is expected during a negative energy balance when lipolysis is stimulated, with the release of fatty acids from stored triglycerides that can then be used for the mitochondrial beta-oxidation within the fat cell or be secreted from the cell to serve as energy source for other tissues. During conditions of energy restriction, an increase of the lipolysis, and intracellular trafficking of fatty acids, results in a decrease in fat mass. This would be in line with the observed correlation between increase of FABP4 and loss of fat mass.

The decreased AldoC during energy restriction is in accordance with previous results in obese subjects on an energy-restricted diet [11, 12]. Concurrently, blood glucose is not changed after energy restriction. The consistently observed decrease in AldoC during energy restriction [13], suggests that it may be a marker for the glycolytic flux in fat tissue. In addition, a parallel between a decrease of glycolytic flux in fat tissue on one hand and a decrease in activity and muscle use on the other hand, may underlie the observed correlation between the decrease in AldoC and the decrease in AEE. The decrease in AEE is in line with previous studies that show a reduction in physical activity following energy restriction [23–28]. Hypothetically, our results could indicate that during reduced glucose availability as a substrate, there is decreased glycolytic flux in fat tissue and decreased activity in order to preserve blood glucose as a supply for glucose-dependent tissues, such as the brain or red blood cells [45]. Additionally, reduced substrate availability may increase the demand for amino acids as an energy source for other tissues [46]. This would be in keeping with the observed correlation between the decrease in AldoC and the decrease in fat free mass.

HADHsc is not significantly increased at the end of the 8-wk VLED, which has been described before [11, 12]. Previously, Bouwman et al. showed a positive correlation between three enzymes of the beta-oxidation (HADHsc, Acetyl-CoA acetyltransferase and Acyl-CoA dehydrogenase) and plasma free fatty acids (FFA) during weight loss maintenance [13]. Apparently, the adipose tissue level of HADHsc parallels the level of plasma FFA. An increased level of FFA during energy restriction would support the energy flux to other peripheral tissues, which could allow lower adaptative thermogenesis in REE. This seems to be in line with the observed correlation between the change in HADHsc and adaptive thermogenesis after the 8-wk VLED. HADHsc is crucial for beta-oxidation [29, 30]. Therefore, it is possible that changes in HADHsc reflect changes in the flux of fatty acids through the mitochondrial beta-oxidation pathway. Hypothetically, up-regulation of the mitochondrial beta-oxidation flux might be the consequence of an activated lipolysis, leading to increased plasma FFA and smaller adaptive thermogenesis in REE.

The correlation between FABP4 and HADHsc would also be in line with the HADHsc level reflecting the lipolytic activity, because this would parallel the requirement for intracellular trafficking of fatty acids. Additionally, an increased trafficking and beta-oxidation of fatty acids in the adipose tissue might coincide with a reduced flux through the glycolytic pathway. In this respect, a positive correlation between AldoC and FABP4 and HADHsc would imply that higher fatty acid flux is better for maintenance of glycolytic flux.

A limitation of this study is the use of total adipose tissue biopsy material for Western blotting, because this could have contained other cell types in the stromal vascular fraction. However, the findings of our previous studies indicate that the majority of the isolated protein is derived from adipocytes [13]. Furthermore, beta-actin showed no significant changes and was chosen as a housekeeping control to be able to compare the present results with those of other studies. Although the selected proteins are involved in the major steps of the glucose and fatty acid metabolism and may reflect the capacity of metabolic pathways, it should be noted that protein levels do not represent the actual flux through the pathways. Furthermore, the observed correlations of the adipose tissues cannot be translated into regulatory mechanisms and are not suited to prove causation. Though, the observed outcomes are consistent with intuitive expectations and the hypothesized mechanisms could be subject of future research.

**Fig. 2:**
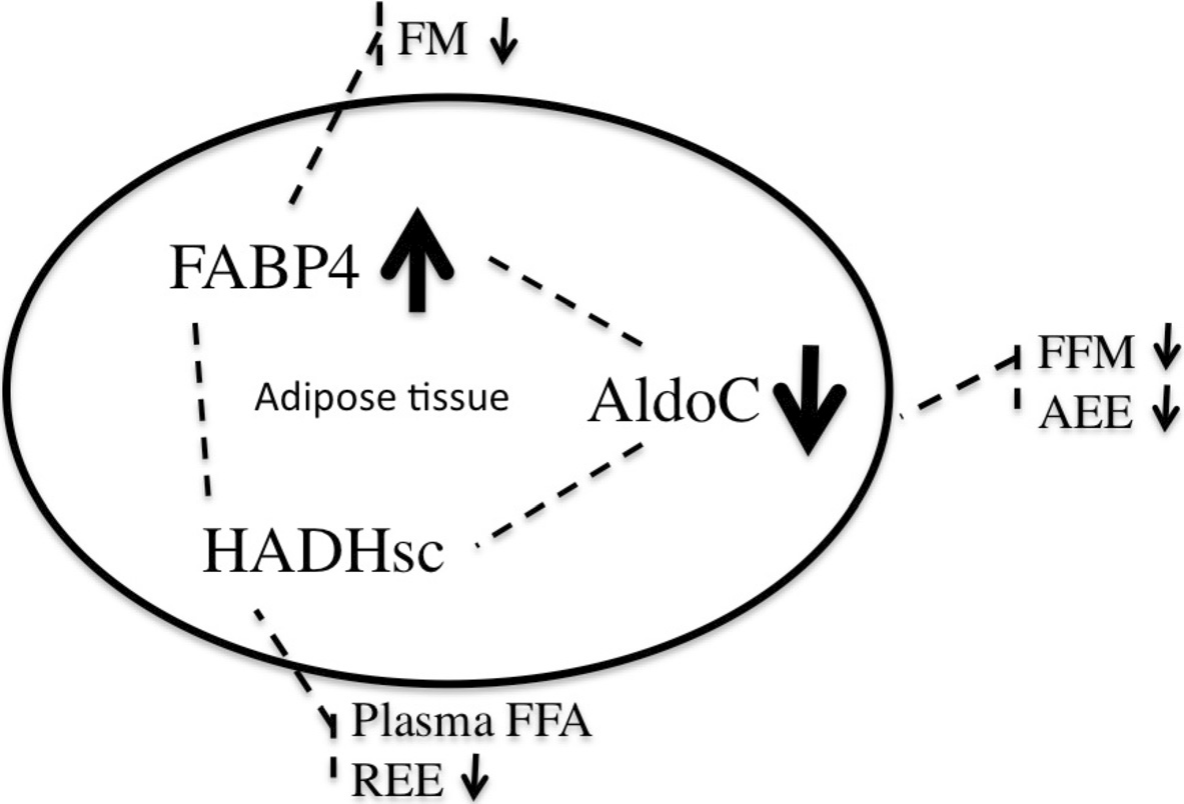
Overview of the network of changes inside the adipose tissue as a result of a negative energy balance and the hypothetical connections with adaptations in body composition and energy expenditure. FABP4, Fatty acid binding protein 4, HADHsc, Short chain 3-hydroxyacyl-CoA dehydrogenase, AldoC, Fructose-bisphosphate aldolase C, FM, fat mass, FFM, fat free mass, FFA, free fatty acids, REE, resting energy expenditure, AEE, activity induced energy expenditure, TEE, total energy expenditure

In conclusion, during energy restriction, the molecular metabolism in adipose tissue is linked to energy expenditure. More specifically, the decrease in AldoC is correlated to the decrease in AEE, which could be explained by the preservation of glucose, and the change in HADHsc is correlated to adaptive thermogensis in REE, which could be explained by changes in the beta-oxidation and lipolysis. Overall, our findings reveal a link between changes on a physiological level and changes of the molecular metabolism in fat cells. This shows the important role of adipose tissue in obese people. The molecular changes in adipose tissue as a result of a negative energy balance might even be the underlying driver of adaptations in body composition and energy expenditure (Fig. 2).
